# Work performance among healthcare workers with post COVID-19 syndrome and its relation to antibody response

**DOI:** 10.1007/s15010-022-01942-4

**Published:** 2022-10-29

**Authors:** Marwa Mohammed Fouad, Nermin Hamdy Zawilla, Lobna Ahmed Maged

**Affiliations:** 1 Department of Occupational and Environmental Medicine, Faculty of Medicine, Cairo University, Cairo, Egypt; 2 Department of Rheumatology and Rehabilitation, Faculty of Medicine, Cairo University, Egypt Cairo,

**Keywords:** Post COVID-19 syndrome, Healthcare workers, Immunological response, Work performance, Presenteeism

## Abstract

**Purpose:**

Health care workers (HCWs) are frontliners in facing Cornoravirus disease (COVID-19) and hence are amongst the high risk groups of acquiring COVID-19 infection. The impact of COVID-19 infection and post-infection sequelae on work performance has deleterious effects on HCWs and the whole community. The aim of the current study is to assess the impact of COVID-19 infection particularly those with post-COVID-19 syndrome on work performance among HCWs and to determine if a possible relationship with antibody response exists.

**Methods:**

A sample of 69 previously PCR-positive health care workers matched to another group of 69 control PCR-negative health care workers from the same clinical departments were subjected to full medical history, clinical examination, measuring serum specific immunoglobulins against severe acute respiratory syndrome coronavirus 2 (SARSCoV-2), Health work performance questionnaire short form of absenteeism and presenteeism and Functional dysfunction grading questionnaire.

**Results:**

The most frequently encountered symptom by patients with post-acute COVID-19 was fatigue while it was dyspnea for those who were chronic COVID patients. Patients with post-acute COVID-19 had a significantly longer time for PCR negative conversion and had a more severe disease. There was no association between post-acute COVID-19 and immunoglobulin positivity. COVID-19 syndrome had a negative impact on work performance manifested by lower relative presenteeism and lower month/year performance ratio (*p* < 0.001, *p* < 0.001). However comparing patients with post-COVID-19 syndrome to patients without the syndrome revealed no significant work performance difference between both groups.

**Conclusion:**

COVID-19 syndrome negatively impacts work performance in HCWs manifested by lower relative presenteeism and lower month/year performance ratio. Although post-COVID-19 results resulted in higher levels of fatigue and functional limitation, it did not have a significant negative impact on work performance. Specific immunoglobulins against SARS CoV-2 were not associated with the post-COVID-19 syndrome.

## Introduction

Cornoravirus disease (COVID-19); the illness caused by Severe acute respiratory syndrome coronavirus 2 (SARS-CoV-2) affected millions of patients worldwide. Symptoms spectrum vary widely starting from asymptomatic disease to life-threatening disease with acute respiratory distress and multi-organ failure. Initially it was considered a short-term illness. Time however started to reveal the long-term effects of the disease including persisting symptoms and lingering damage giving rise to terms such as post-COVID-19 syndrome and long COVID [1].

Post-COVID-19 syndrome seems to be a multisystem disease, sometimes occurring after a relatively mild acute illness. In the absence of agreed definitions, post-acute COVID-19 was defined by some as symptoms extending beyond three weeks from the onset of first one and chronic COVID-19 as symptoms extending beyond 12 weeks [2].

Persistent viremia due to weak or absent antibody response, relapse or reinfection [3], inflammatory and other immune reactions [4], and mental factors such as post-traumatic stress [5] may all contribute for the persistent symptoms.

Virus specific antibodies represent a valuable serological maker. Their role in the pathogenesis, control of viral replication and clinical course of the disease is still a matter of debate with some studies demonstrating their role in viral neutralization and clearance, other studies showed an association with more severe clinical cases with theories suggesting a potential role in pulmonary pathology [6]. Their role in post-COVID-19 syndrome was not thoroughly investigated.

Return to work policy for health care workers (HCWs) according to the Center for Disease Control and Prevention (CDC) based on either a testing-based approach with two negative nasopharyngeal swabs taken 24 h apart; or a non-testing-based approach based on symptom resolution so that at least 3 days have passed free of fever since recover and, at least 10 days have passed since symptoms first appeared [7]. Persistent symptoms in the setting of post-COVID-19 syndrome do not interfere with the return to work policy currently followed.

Absenteeism and presenteeism are used to assess work performance and are frequently used in clinical trials where absenteeism is a condition when workers do not show at work on the working day, which may be caused by any condition whereas presenteeism is when workers come to work despite feeling sick [8]. The tendency to continue working despite feeling unwell may be because of heavy workloads, shift work and irreplaceable duties [9] and the COVID-19 pandemic resulted in an intense work overload for HCWs. Absenteeism and presenteeism among HCWs have been widely studied [10, 11] but not as a part of the current pandemic in post-COVID-19 syndrome patients.

The limited studies about the post-COVID-19 syndrome immunological response and its relation to the persistent symptoms together with its effect on absenteeism and presenteeism encouraged us to perform the current study aiming to assess the impact of COVID-19 infection particularly those with post-COVID-19 syndrome on work performance among HCWs and to determine if a possible relationship with antibody response exists.

## Methods

A case control study was conducted on 140 HCWs of which 71 had history of polymerase chain reaction (PCR) proven COVID-19 infection whose PCR converted negative and returned to work at least 1 month before the study. Sixty-nine PCR-negative HCWs control who were tested as they contacted positive cases yet had no symptoms were recruited from the same clinical departments in Cairo University Hospitals. Both groups included doctors, nurses, workers and technicians who are in direct contact with patients. Two HCWs were excluded from the COVID-19 positive group as they had bronchial asthma owing to difficulty of differentiation between asthma, COVID-19 and post-COVID-19 symptoms. The 69 cases were further classified according to the persistence of symptoms into post-acute COVID-19 patients who had symptoms persistent for more than 3 weeks and chronic COVID-19 patients whose symptoms persisted for more than 3 months [2]. The study was conducted between May and September 2020.

Since there is no available data regarding assessment of work performance among post-COVID HCWs, a pilot study was conducted to assess work performance in post-COVID HCWs compared to control. Based on its results, comparing post-COVID HCWs to control with ratio 1:1, the median relative presenteeism in post-COVID HCWs was 0.89 with standard deviation (SD) 0.09 while the median relative presenteeism in control group was 1 with SD 0.0125, so 18 participants per group were needed, compensated by 15% due to the use of nonparametric tests, and compensated by 30% for suspected losses so the final sample size includes 30 subject per group (total 60 participant) to be able to reject the null hypothesis that the population means relative presenteeism in post-COVID HCWs and their control are equal with probability (power) 0.99. The Type I error probability associated with test of this null hypothesis was 0.05. Sample size was calculated using G power program.

Patients were offered a comprehensive medical assessment with detailed history (including personal, present, past, family history in addition to occupational history) and complete clinical examination for detection of clinical signs of comorbidities. Data on specific symptoms potentially related to COVID-19 were obtained using a standardized questionnaire administered at enrollment and a detailed history of hospital admission, requirement of ventilatory support and CT chest performed during admission were obtained from patients records and used to grade disease severity where patients with mild clinical symptoms and negative imaging were classified as having mild COVID-19; those with fever, respiratory symptoms and pneumonic manifestation in CT chest were classified as having moderate COVID-19 whereas patients with any of the following: respiratory rate ≥ 30 times/min; resting fingertip oxygen saturation ≤ 93%; arterial partial pressure of oxygen (PaO_2_)/fraction of inspired oxygen (FiO_2_) ≤ 300 mmHg; pulmonary imaging shows significant progression of lesions > 50% within 24–48 h were classified as having severe COVID-19 [12]. Patients were asked to retrospectively recount the presence or absence of COVID-19 symptoms at 3 weeks and 3 months from onset of symptoms and if any symptoms are still persistent at the time of the visit.

### Questionnaires

A face to face interview was done where the questionnaires were explained and directly asked by the investigators.

- **Health and work performance questionnaire short form of absenteeism and presenteeism:**
  World Health Organization Health and Work Performance Questionnaire (WHO‐HPQ) is a self‐report questionnaire used to measure job performance [13]. We used the WHO‐HPQ short form that focuses on two aspects: presenteeism and absenteeism. Absolute presenteeism measured by the question: “On a scale from 0 to 10, where 0 is the worst job performance anyone could have at your job and 10 is the performance of a top worker, how would you rate your overall job performance on the days you worked during the past four weeks?” represents actual performance and is used as a measure of work productivity; whereas Relative presenteeism is a ratio of actual performance to the performance of most workers at the same job. The score ranges from 0.25 to 2.0, where 0.25 signifies the worst and 2.0 the best relative performance. It was obtained by dividing the response of question “Using the same 0-to-10 scale, how would you rate your overall job performance on the days you worked during the past 4 weeks (28 days)?” divided by the response of the previous question of absolute presenteeim. Absenteeism is scored in terms of hours lost per month. The measure of absolute absenteeism is expressed in raw hours, with a negative lower bound (if the person works more than expected) and an upper bound equal to the number of hours the respondent is expected to work. The measure of relative absenteeism is expressed as a percentage of expected hours.
  In addition to the previously mentioned scores we obtained a ratio of performance over the 1st month following return to work after COVID-19 to the performance over the past year. In controls the ratio of performance in the month following negative PCR to the performance over last year was obtained. This was obtained by dividing the results of the question “On a scale from 0 to 10, where 0 is the worst job performance anyone could have at your job and 10 is the performance of a top worker, how would you rate your overall job performance on the days you worked during the past 4 weeks?” by the results of the question “Using the same 0-to-10 scale, how would you rate your usual job performance over the past year or two?”.
- **Post-COVID-19 functional status questionnaire**
  Functional status was assessed according to the post-COVID-19 functional status scale, which consists of an ordinal scale for assessment of patient-relevant functional limitations. Grade 0 reflects the absence of any functional limitation, grade 1: negligible limitations with persistent symptoms but has no effect on everyday life, grade 2: limitations in everyday life, occasionally need to avoid or reduce usual activities, grade 3: limitations in everyday life and the patient is not able to perform all usual activities, grade 4: severe functional limitations requiring assistance with activities of daily living [14].

### Laboratory investigations

SARS CoV-2 immunoglobulins IgG/IgM were measured using QuickZen COVID-19 IgM/IgG Kit (QuickZen) (ZenTech, Angleur, Belgium), an immune colloidal gold technique intended for the qualitative detection of IgG and IgM against SARS-CoV-2 in human whole blood, serum or plasma specimens. The reagent-binding pad is coated with colloidal gold-labelled recombination antigen and rabbit IgG antibodies serve as control.

Blood sample of 2 cm of blood was obtained by venipuncture under aseptic conditions on plain tube and centrifugated for serum separation where 10 µl of serum was obtained for the rapid kit and results were interpreted 10 min later.

An informed consent to participate in the study was obtained after explaining the importance of the study. The study was approved by the Research Ethics Committee, Faculty of Medicine, Cairo University (REC n-75-2021). It has been performed in accordance with the ethical standards laid down in the 1964 Declaration of Helsinki and its later amendments.

### Statistical analysis

Data were coded and entered using the statistical package for the Social Sciences (SPSS) version 26 (IBM Corp., Armonk, NY, USA). Data was summarized using mean, standard deviation, median, minimum and maximum in quantitative data and using frequency (count) and relative frequency (percentage) for categorical data. Comparisons between quantitative variables were done using the non-parametric Kruskal–Wallis and Mann–Whitney tests. For comparing categorical data, Chi square (*χ*^2^) test was performed. Exact test was used instead when the expected frequency is less than 5. *P* values less than 0.05 were considered as statistically significant.

## Results

The current study was performed on Cairo University hospitals personnel included 69 PCR proven COVID-19 HCWs with mean age 39.4 ± 9.6 years and 69 PCR negative HCWs who served as controls with mean age 43 ± 11.2 years. The majority of the studied population were non-smokers, where only 8 (11.6%) were smokers among the PCR positive individuals and 17 (24.6%) were smokers among PCR negative individuals. The demographic features, use of personal protective equipment, co-morbidities and the vaccination status of the studied groups showed no statistically significant difference (Table 1). The clinical features of COVID-19 positive patients are also illustrated in Table 1.

**Table 1:** Demographic and clinical characteristics of the studied population:

	PCR positive patients *N* = 69	PCR negative controls *N* = 69	*p* value
Sex, *N* (%)			
Male	40 (58.0%)	31 (44.9%)	0.13
Female	29 (42.0%)	38 (55.1%)	
BMI, Median (IQ range)	29.4 (25.8–35.3)	27.8 (24.2–32.5)	0.18
Occupation, *N* (%)			
Doctor	10 (14.5%)	14 (20.3%)	0.64
Nurses	34 (49.3%)	27 (39.1%)	
Workers	18 (26.1%)	21 (30.4%)	
Others	7 (10.1%)	7 (10.1%)	
Department, *N* (%)			
Medical	46 (66.7%)	55 (79.7%)	0.22
Surgical	16 (23.2%)	10 (14.5%)	
ICU	7 (10.1%)	4 (5.8%)	
Smoking index pack/year, Median (IQ range)	13.75 (6.25–23.25)	13.75 (5–29.5)	0.80
Use of personal protective equipment, *N* (%)			
Masks	55 (79.7%)	54 (78.3%)	0.83
Face shield	5 (7.2%)	8 (11.6%)	0.44
Gloves	6 (8.7%)	9 (13%)	0.41
Gown	7 (10.1%)	8 (11.6%)	0.78
Co-morbidities, *N* (%)			
Any co-morbidity	17 (24.6%)	17 (24.6%)	1
Diabetes Mellitus	9 (13.0%)	6 (8.7%)	0.41
Hypertension	8 (11.6%)	7 (10.1%)	0.78
Cardiovascular disease	1 (1.4%)	1 (1.4%)	1
Chronic lung disease	2 (2.9%)	2 (2.9%)	1
Chronic liver disease	0 (0%)	0 (0%)	–
Chronic kidney disease	0 (0%)	0 (0%)	–
Cancer	0 (0%)	1 (1.4%)	1
Thyroid disease	1 (1.4%)	0 (0%)	1
Autoimmune disease	1 (1.4%)	2 (2.9%)	1
Vaccination status, *N* (%)			
Tuberculosis vaccine	24 (34.8%)	16 (23.2%)	0.13
Pneumococcal vaccine	1 (1.4%)	0 (0%)	1
Seasonal Influenza vaccine	5 (7.2%)	1 (1.4%)	0.21
CT chest, *N* (%)			
Pneumonia	30 (43.5%)		
Normal	27 (39.1%)		
Not done	12 (17.4%)		
Severity of COVID-19, *N* (%)			
Mild	14 (20.3%)		
Moderate	50 (72.5%)		
Severe	5 (7.2%)		
Ventilatory support, *N* (%)			
Oxygen need	5 (7.2%)		
Mechanical ventilation	0 (0%)		
Medications, *N* (%)			
Anti-retroviral	1 (1.4%)		
Hydroxychloroquine	51 (73.9%)		
Azithromycin	64 (92.8%)		
Tocilizumab	5 (7.2%)		
Anticoagulant	8 (11.6%)		
Post-COVID-19 complications, *N* (%)			
Any complication	6 (8.6%)		
Acute cardiac injury	0 (0%)		
Acute renal injury	3 (4.3%)		
Acute liver injury	3 (4.3%)		
Stroke	0 (0%)		
Acute pulmonary embolism	1 (1.4%)		
Immuoglobulin, *N* (%)			
Negative	44 (63.8%)		
IgM	10 (14.5%)		
IgG	9 (13.0%)		
Both (IgM, IgG)	6 (8.7%)		

*COVID* Corona virus, *PCR* polymerase chain reaction, *BMI* Body mass index, *IQ* Interquartile, *ICU* Intensive care unit, *CT* Computed tomography, *IgM* Immunoglobulin M, *IgG* Immunoglobulin G

In descending order of frequency the most frequently encountered symptoms suffered by COVID-19 patients in the acute COVID-19 phase were sleep disturbance, difficulty in concentration and abdominal pain (89.9%, 81.2% and 78.3% respectively). Fifty-two (75.4%) patients were classified as having post-acute COVID-19. The most frequently encountered symptoms by patients with post-acute COVID-19 were fatigue, joint/muscle aches, anxiety, depression and sleep disturbance (49.3%, 47.8%, 39.1%, 39.1% and 39.1% respectively) whereas 37 (24.6%) patients were classified as having chronic COVID-19. The symptoms most frequently encountered by chronic COVID-19 patients were dyspnea, joint/muscle aches, fatigue and palpitation (15.9%, 14.5%, 14.5 and 14.5% respectively) (Table 2).

**Table 2:** Symptoms frequency and duration among COVID-19 patients

Symptom	Acute COVID-19 infection *N* = 69 *N* (%)	Acute post-COVID-19 *N* = 52 *N* (%)	Chronic COVID-19 *N* = 37 *N* (%)	Duration of symptom (days) Median (IQ range)
Sleep disturbance	62 (89.9%)	27 (39.1%)	7 (10.1%)	30 (7–50)
Difficulty in concentration	56 (81.2%)	17 (24.6%)	8 (11.6%)	30 (10–90)
Abdominal pain	54 (78.3%)	2 (2.9%)	1 (1.4%)	8 (4.5–14)
Vomiting	50 (72.5%)	26 (37.7%)	0 (0%)	7 (2–20)
Diarrhea	47 (68.1%)	7 (10.1%)	0 (0%)	7 (3–14)
Nausea	47 (68.1%)	3 (4.3%)	0 (0%)	7 (3–14)
Loss of taste	45 (65.2%)	16 (23.2%)	1 (1.4%)	17 (7–30)
Dizziness	45 (65.2%)	13 (18.8%)	1 (1.4%)	14 (7–30)
Dyspnea	44 (63.8%)	26 (37.7%)	11 (15.9%)	30 (14–86.3)
Headache	44 (63.8%)	14 (20.3%)	3 (4.3%)	14 (7–25)
Loss of appetite	44 (63.8%)	16 (23.2%)	2 (2.9%)	19 (7–30)
Red eye	42 (60.9%)	6 (8.7%)	1 (1.4%)	17 (7–30)
Skin rash	40 (50.8%)	2 (2.9%)	1 (1.4%)	10 (7–30.8)
Dry skin	39 (56.5%)	0 (0%)	0 (0%)	12 (10–30)
Dry mouth	38 (55.1%)	11 (15.9%)	0 (0%)	14 (5.5–30)
Dry eye	35 (50.7%)	5 (7.2%)	1 (1.4%)	30 (14–30)
Joint/muscle aches	32 (46.4%)	33 (47.8%)	10 (14.5%)	30 (10–60)
Fatigue	30 (43.5%)	34 (49.3%)	10 (14.5%)	30 (10–60)
Rhinitis	29 (42%)	3 (4.3%)	0 (0%)	7 (3–12)
Anosmia	23 (33.3%)	16 (23.2%)	3 (4.3%)	14 (5–30)
Fever	21 (30.4%)	1 (1.4%)	0 (0%)	5 (2–10)
Palpitation	19 (27.5%)	18 (26.1%)	10 (14.5%)	30 (7–112.5)
Chest pain	16 (23.2%)	6 (8.7%)	1 (1.4%)	20 (5–30)
Expectoration	12 (17.4%)	8 (11.6%)	1 (1.4%)	30 (14–30)
Cough	11 (15.9%)	17 (24.6%)	2 (2.9%)	17 (7–30)
Sore throat	10 (14.5%)	9 (13%)	2 (2.9%)	10 (3.8–23.3)
Hallucination/night mares	9 (13%)	4 (5.8%)	2 (2.9%)	15 (7–30)
Depression	7 (10.1%)	27 (39.1%)	9 (13%)	30 (14.3–60)
Anxiety	7 (10.1%)	27 (39.1%)	6 (8.7%)	30 (14–60)

Patients with post-acute COVID-19 had a significantly longer time for PCR negative conversion, were more likely to have CT proven pneumonia and had a more severe disease (*p* = 0.004, 0.024 and 0.001,  respectively). The use of Azithromycin was negatively associated with the development of post-acute COVID-19 (*p* = 0.012). There was no association between post-acute COVID-19 and immunoglobulin positivity (Table 3). Patients with positive immunoglobulins had a mean duration of 88.3 ± 31.2 days since their PCR converted negative in comparison to 105.9 ± 34.8 days in patients with negative immunoglobulins (untabulated data).

**Table 3:** Comparing post-acute COVID-19 patients to the non-post-acute COVID-19 among the studied COVID-19 patients

	Post-acute COVID-19 patients *N* = 52	Non post-acute COVID-19 patients *N* = 17	*p* value
Sex, *N* (%)			
Male	33 (63.5%)	7 (41.2%)	0.11
Female	19 (36.5%)	10 (58.8%)	
Age, Mean ± SD	39.25 ± 9.6	39.9 ± 10	0.96
BMI, Median (IQ range)	29.4 (26–35.2)	32 (24.7–36.3)	0.31
Occupation, *N* (%)			
Doctor	5 (9.6%)	5 (29.4%)	0.11
Nurses	27 (51.9%)	7 (41.2%)	
Workers	13 (25%)	5 (29.4%)	
Others	7 (13.5%)	0 (0%)	
Smoking, *N* (%)	6 (11.5%)	2 (11.8%)	1
Smoking index (Median)	11.3	20	0.4
Co-morbidities, *N* (%)			
Diabetes mellitus	6 (11.5%)	3 (17.6%)	0.68
Hypertension	7 (13.5%)	1 (5.9%)	0.67
Cardiovascular disease	1 (1.9%)	0 (0%)	1
Chronic lung disease	2 (3.8%)	0 (0%)	1
Thyroid disease	1 (1.9%)	0 (0%)	1
Autoimmune disease	1 (1.9%)	0 (0%)	1
Vaccination status, *N* (%)			
Tuberculosis vaccine	17 (32.7%)	7 (41.2%)	0.52
Pneumococcal vaccine	1 (1.9%)	0 (0%)	1
Seasonal Influenza vaccine	5 (9.6%)	0 (0%)	0.32
PCR conversion in days, Median (IQ range)	38.5 (29.3–52.8)	16 (11.5–36.5)	0.004*
CT chest, *N* (%)			
Pneumonia	27 (51.9%)	3 (17.6%)	0.024*
Normal	16 (30.8%)	11 (64.7%)	
Not done	9 (17.3%)	3 (17.6%)	
Severity of COVID-19, *N* (%)			
1	5 (9.6%)	9 (52.9%)	0.001*
2	42 (80.8%)	8 (47.1%)	
3	5 (9.6%)	0 (0%)	
Ventilatory support, *N* (%)			
Oxygen need	5 (9.6%)	0 (0%)	0.32
Medications, *N* (%)			
Anti-retroviral	1 (1.9%)	0 (0%)	1
Hydroxychloroquine	42 (80.8%)	9 (52.9%)	0.053
Azithromycin	51 (98.1%)	13 (76.5%)	0.012*
Tocilizumab	5 (9.6%)	0 (0%)	0.32
Anticoagulant	7 (13.5%)	1 (5.9%)	0.67
Post-COVID-19 complications, *N* (%)			
Acute renal injury	3 (5.8%)	0 (0%)	0.57
Acute liver injury	3 (5.8%)	0 (0%)	0.57
Acute pulmonary embolism	1 (1.9%)	0 (0%)	1
Immuoglobulin, *N* (%)			
Negative	35 (67.3%)	9 (52.9%)	0.21
IgM	6 (11.5%)	4 (23.5%)	
IgG	8 (15.4%)	1(5.9%)	
Both (IgM, IgG)	3 (5.8%)	3 (17.6%)	

Severity of COVID-19 was classified according to the WHO classification [12]

*COVID* Corona virus, *SD* standard deviation, *BMI* body mass index, *IQ* interquartile, *ICU* intensive care unit, *PCR* polmerase chain reaction, *CT* computed tomography, *IgM* immunoglobulin M, *IgG* immunoglobulin G

*Statistically significant *p* value < 0.05

Patients with COVID-19 syndrome had significantly higher functional limitation and fatigue (*p* < 0.001). They also had significantly lower relative presenteeism and lower work performance in the month following PCR COVID-19 negative testing in comparison to last year (*p* < 0.001) (Table 4).

**Table 4:** Functional limitations, Fatigue scale, World Health Organization’s Health and Work Performance Questionnaire (WHO-HPQ) short form of absenteeism and presenteeism questions among COVID-19 PCR positive patients and negative controls

	COVID-19 PCR positive patients *N* = 69	COVID-19 PCR negative control *N* = 69	*p* value
Functional limitation, Median (IQ range)	1 (0–2)	0 (0–0)	< 0.001*
Fatigue scale, Median (IQ range)	6 (0–6)	0 (3–8)	< 0.001*
WHO-HPQ questionnaire (short form)			
* Over the past 4 weeks did you miss an entire work day because of problems with your physical or mental health,* Median (IQ range)	0 (0–0)	0 (0–0)	0.08
* Over the past 4 weeks did you miss an entire work day for any other reason (including vacation)¸* Median (IQ range)	0 (0–0)	0 (0–0)	0.008*
* Over the past 4 weeks did you miss part of a work day because of problems with your physical or mental health*, Median (IQ range)	0 (0–0)	0 (0–0)	0.23
* Over the past 4 weeks did miss part of a work day for any other reason (including vacation*), Median (IQ range)	0 (0–0)	0 (0–0)	0.98
* Over the past 4 weeks did you come in early, go home late, or work on your day off*, Median (IQ range)	0 (0–0)	0 (0–0)	0.16
* On a scale 0-to-10 scale, how would you rate your usual job performance over the past year or two*, Mean ± SD	8.91 ± 1.2	8.8 ± 1.1	0.47
* On a scale 0-to-10 scale, how would you rate your overall job performance on the days you worked during the past 4 weeks (28 days),* Mean ± SD	7.10 ± 2	8.8 ± 1.1	< 0.001*
Relative absenteeism, Median (IQ range)	0 (0–0)	0 (0–0)	0.08
Relative presenteeism, Median (IQ range)	0.89 (0.65–1)	1 (1–1.05)	< 0.001*
Ratio of performance in month following PCR COVID-19 negative testing to last year ratio, Mean ± SD	0.8 ± 0.2	1 ± 0.08	< 0.001*

*COVID-19* Corona virus, *PCR* polmerase chain reaction, *IQ* interquartile range, *WHO-HPQ* World Health Organization’s Health and Work Performance Questionnaire

*Statistically significant *p* value < 0.05

Patients with post-acute COVID-19 had significantly higher functional limitation in comparison to patients without the condition (*p* < 0.001) and in spite of having lower work performance in the month following PCR COVID-19 negative testing in comparison to last year and a lower relative presenteeism, the results did not reach statistical significance (*p* = 0.06, *p* = 0.15 respectively). Relative absenteeism was similar in both groups (Table 5).

**Table 5:** Functional limitations, fatigue scale, World Health Organization’s Heath and Work Performance Questionnaire (HPQ) short form of absenteeism and presenteeism questions among acute post-COVID-19 and non-post-acute COVID-19 patients

	Post-acute COVID-19 patients *N* = 52	Non Post-acute COVID-19 patients *N* = 17	*p* value
Functional limitation, Median (IQ range)	2 (0–2)	1 (1–1)	< 0.001*
Fatigue scale, Mean ± SD	5.98 ± 2.31	3.32 ± 1	< 0.001*
WHO-HPQ questionnaire (short form)			
* Over the past 4 weeks did you miss an entire work day because of problems with your physical or mental health,* Median (IQ range)	0 (0–0)	0 (0–0)	0.11
* Over the past 4 weeks did you miss an entire work day for any other reason (including vacation)¸* Median (IQ range)	0 (0–0)	0 (0–0)	0.13
* Over the past 4 weeks did you miss part of a work day because of problems with your physical or mental health*, Median (IQ range)	0 (0–0)	0 (0–0)	0.19
* Over the past 4 weeks did miss part of a work day for any other reason (including vacation*), Median (IQ range)	0 (0–0)	0 (0–0)	0.32
* Over the past 4 weeks did you come in early, go home late, or work on your day off*, Median (IQ range)	0 (0–0)	0 (0–0)	0.42
* On a scale 0-to-10 scale, how would you rate your usual job performance over the past year or two*, Mean ± SD	7 ± 1.9	7.8 ± 1.9	0.3
* On a scale 0-to-10 scale, how would you rate your overall job performance on the days you worked during the past 4 weeks (28 days),* Mean ± SD	9 ± 1.1	8.6 ± 1.4	0.19
Relative absenteeism, Median (IQ range)	0 (0–0)	0 (0–0)	0.27
Relative presenteeism, Mean ± SD	0.8 ± 0.3	1 ± 0.2	0.15
Ratio of performance in month following PCR COVID-19 negative testing to last year ratio, Mean ± SD	0.78 ± 0.2	0.9 ± 0.18	0.06

*COVID-19* Corona virus, *IQ* interquartile range, *WHO-HPQ* World Health Organization’s Health and Work Performance Questionnaire

*Statistically significant *p* value < 0.05

## Discussion

The impact of COVID-19 infection and post-infection sequelae on work performance has deleterious effects not on HCWs alone but on the whole community. A global approach to protect HCWs from infection and awareness of both the acute and long-term complications of the disease are of utmost importance [15].

In the current study fatigue was the most common symptom experienced by HCWs with post-acute- COVID-19. were suffering from fatigue most commonly. Immunoglobulin positivity was not associated to post- acute-COVID-19 syndrome in the current study. Patients with post-acute COVID-19 had significantly higher functional limitation and in spite of having lower month/year work performance and relative presenteeism, the results didn’t reach statistical significance. Post-COVID-19 syndrome gained public attention and was the focus of a number of studies [16–23] and was even declared a disability under the Americans with Disabilities Act [24]. Patients with all grades of COVID-19 severity were recruited ranging from asymptomatic to severe and some studies focused on ICU admitted patients. Its incidence among different studies varied from 30% in one study conducted on 177 patients from USA [16] to 87.4% in a study focused on patients discharged from ICU in Italy with 55.2% of patients suffering from ≥ 3 symptoms [17]. Duration of follow up was also variable; studies reported that symptoms ranged from 1 to beyond 12 months post discharge [16–23]. In concordance with the current study fatigue is the most frequently encountered symptom in most studies addressing post-COVID-19 syndrome with an incidence reaching 72% with resultant significant disability and decline in quality of life [17, 19, 22]. Dyspnea is another common symptom with incidence ranging from 11.1 to 71% [1, 17–19, 21].

Studies focused on HCWs showed similar results [25–27]. Reported incidence of post-COVID-19 syndrome reached 73% in a study including 2053 participants working in health care and social services in Germany. Fatigue was the most commonly reported symptom followed by concentration/memory problems and shortness of breath [27]. In concordance with the current study findings, severity of acute COVID-19 infection was associated with the development of post-COVID-19 syndrome. Although the pathogenesis of post-COVID-19 syndrome remains largely unknown, potential mechanisms include direct viral related mechanisms, immune dysregulation and post-critical illness sequaleae [28]. Profound prolonged inflammation and immune dysregulation with exaggerated inflammatory response are among the most acceptable theories. Elevated level of cytokines particularly IL-6 was implicated in a wide array of manifestations including fatigue, anxiety, depression, olfactory dysfunction, orthostatic hypotension as well as pulmonary fibrosis. IL-6 has well-known inflammatory and autoimmune properties [29]. Pulmonary fibrosis is one of the most worrisome post-COVID-19 complications with both viral and immune mediated mechanisms contributing to epithelial-endothelial damage and subsequent fibrosis [30]. Lung affection is not only implicated in post-COVID-19 respiratory symptoms but was also proposed to be involved of pathogenesis of other symptoms as fatigue [22].

The present study did not find any association between post-COVID-19 syndrome and immunoglobulin positivity. A potential explanation is the wide variation of the time period between COVID-19 infection and immunoglobulin testing in the patients recruited. Immunoglobulin testing was done at least 29 days and up to 197 days following COVID-19 infection. Disease severity is another factor that could have contributed to such findings where only 7.2% of our cohort had severe COVID-19 infection. Patients with mild symptoms develop a weaker and less lasting immune response to the virus, with a decrease in the level of antibodies 2–3 months of infection [31]. Although IL-6 has an important role in immunoglobulin production it was not found to correlate with immunoglobulins [32].

In the current study patients with COVID-19 disease had significantly higher functional limitation and significantly lower relative presenteeism and lower month/year performance. Patients with post-COVID-19 syndrome had significantly higher functional limitation and a non-significant lower month/year performance in comparison to patients without post-COVID-19 syndrome. In accordance with the current study a large proportion of hospitalized patients with COVID-19 had diminution of the functional status 6 months after hospitalization [33].

Although absenteeism and presenteeism and its effect on work performance among health care workers were previously studied [10, 11]**,** in the group of HCWs with post-COVID-19 syndrome there has been limited studiest and to the best of our knowledge WHO-HPQ was not previously performed in this group. Disruption of work life was demonstrated in 8% of HCWs experiencing long-term symptoms bearing in mind the study population had mild COVID-19 [20]. Although 32% of HCWs were struggling to cope with post-COVID-19 symptoms only 2% took sick leave in another study [26].

The prevalence of presenteeism was 77% in one study including 120 nurses and respiratory therapists. Approximately 38% of participants responded that working during the pandemic negatively impacted their emotional health whereas 40% reported a negative impact on both their physical and emotional health [34]. Prevalence reached 98.2% in a study conducted on 503 Egyptian nurses. Participants attributed organizational factors such as fear of disciplinary action, staff shortages and limited pay for absenteeism and personal factors such as job insecurity, shortage of alternative job opportunities and professional obligation to the community as likely causes [35].

Another study assessing COVID-19 patients following hospital discharge of which 22% of patients were HCWs and 32% were admitted to ICU found that 40% of patients returned to the same level of employment, 5% converted from full-time to part-time employment whereas 37.5% of patients were on sick leave 29–71 days post-discharge. Patients experienced significant worsening of quality of life and the study suggested the need of implementation of post-discharge rehabilitation of COVID-19 patients [19]. Significant reduction of quality of life was demonstrated 100 days following admission with 69.1% of patients returning to work at time of assessment [18]. Assuming that with the fear of contracting COVID-19, the likelihood of working while ill is lower. However, COVID-19 pandemic highlights the dangers of previous practices of working when sick particularly among HCWs where it was demonstrated to impact the quality of care and resulted in staff-to-patient transmission, medication errors, financial losses to organizations, and employee burnout [34, 36].

Another study addressing university staff and students reported 7% absenteeism in staff and 10% in students and presenteeism in 26% of staff and 40% of the students. The study also reported anxiety and depression in 22–24% of staff and 37.2% and 46.5% of students. Younger age, chronic medical illness both organic and functional, social isolation and low exercise level were identified as predictors of both absenteeism and presenteeism [37]. A recent meta-analysis attributed worsening of work performance and absenteeism not only as direct outcomes of the pandemic but also to poor mental health, anxiety and depression [38]; factors exaggerated by post-COVID-19 syndrome.

In conclusion, work performance can be affected among HCWs with COVID-19 syndrome. Specific COVID-19 immunoglobulins were not associated with the post-COVID-19 syndrome. Although patients with post-COVID-19 syndrome had higher levels of fatigue and functional limitation, it did not have a significant negative impact on their work performance in comparison to patients without the condition.

The present study has some limitations. The cross-sectional study design may impede the conclusion cause-effect relationship. The data about absenteeism, presenteeism, work performance and the persistent post-COVID-19 symptoms, were collected using a self-reported questionnaire, which may be subjected to recall bias. Self-assessment work performance may possibly be at a variance depending on the personality of each individual. Workers may overrate themselves or underestimate others. However, this study used a standardized questionnaire, which might minimize the bias to a certain degree. The characteristics of serological immunoassay tests, which are currently not sufficiently explored and validated is another limitation. Most of the tests are still not validated, and, only manufacturer data about test performance is available.

Although the situation of COVID-19 has markedly improved in comparison to the earlier waves, post-COVID-19 syndrome continues to be a problem with deleterious effects on productivity. We recommend studying the effect of post-COVID-19 syndrome on work performance on a larger scale and the performance on studies focused on development of coping mechanisms and rehabilitation strategies particularly among HCWs.

## Data Availability

Certain data will be shared on reasonable request to the corresponding author with permission of The Research Ethics Committee, Faculty of Medicine, Cairo University.
